# IDENTIFYING HOUSING PREFERENCES IN AN AGE-FRIENDLY COMMUNITY

**DOI:** 10.1093/geroni/igz038.933

**Published:** 2019-11-08

**Authors:** Natalie Pope, Allison Gibson

**Affiliations:** University of Kentucky, Lexington, Kentucky, United States

## Abstract

More than a decade ago, the World Health Organization’s (WHO) age-friendly cities and communities program was established to guide communities in preparing for rapid population aging. WHO identified eight domains of livability that influence health and wellbeing of older adults—housing, one of the eight domains is the focus of this study. Partnering with our local Age Friendly initiative, we examined housing preferences and values for residents of a mid-sized urban community. A cross-sectional survey was administered online via Qualtrics and through face-to-face data collection across the community (e.g., public libraries, community farmers’ markets). Participants aged 30+ completed a researcher-devised survey about housing-related preferences and values. Data were analyzed using descriptive and inferential statistics. Of our 518 respondents, half were 60+ and most were women (73.2%). Younger respondents (age 30-59) were more likely to be married or living with a partner (73.7%). There was little difference in housing preferences between older and younger respondents or among those with varying financial means. Analysis revealed the top five housing considerations were: “safety” (78.8%), “affordability” (73.2%), “privacy” (51.9%), “proximity to services I frequently use” (50.6%), and “accessibility” (42.4%). An unexpected finding was that almost 50% of older respondents expressed a willingness to share a home with a roommate. Data suggests that city planners and property developers should prioritize these preferences when planning for the housing related needs of older residents. Alternative housing models, such as cohousing, should be explored further. Future research should examine interpretation of such housing preferences (i.e., what’s considered affordable?).

